# MultiTest V.1.2, a program to binomially combine independent tests and performance comparison with other related methods on proportional data

**DOI:** 10.1186/1471-2105-10-443

**Published:** 2009-12-23

**Authors:** Thierry De Meeûs, Jean-François Guégan, Anatoly T Teriokhin

**Affiliations:** 1 IRD, UMR 177 IRD-CIRAD "Trypanosomoses", Centre International de Recherche-Développement sur l'Elevage en zone Subhumide (CIRDES), 01 BP 454, Bobo-Dioulasso 01, Burkina-Faso; 2 Génétique et Evolution des Maladies Infectieuses, UMR 2724 CNRS/IRD/Université de Montpellier, and French School of Public Health, 911 Av Agropolis, BP 64501, 34394 Montpellier Cedex 5, France; 3 Section of General Ecology, Dept of Biology, Moscow Lomonosov State University, Moscow 119899, Russia

## Abstract

**Background:**

Combining multiple independent tests, when all test the same hypothesis and in the same direction, has been the subject of several approaches. Besides the inappropriate (in this case) Bonferroni procedure, the Fisher's method has been widely used, in particular in population genetics. This last method has nevertheless been challenged by the SGM (symmetry around the geometric mean) and Stouffer's *Z*-transformed methods that are less sensitive to asymmetry and deviations from uniformity of the distribution of the partial *P*-values. Performances of these different procedures were never compared on proportional data such as those currently used in population genetics.

**Results:**

We present new software that implements a more recent method, the generalised binomial procedure, which tests for the deviation of the observed proportion of *P*-values lying under a chosen threshold from the expected proportion of such *P*-values under the null hypothesis. The respective performances of all available procedures were evaluated using simulated data under the null hypothesis with standard *P*-values distribution (differentiation tests). All procedures more or less behaved consistently with ~5% significant tests at *α *= 0.05. Then, linkage disequilibrium tests with increasing signal strength (rate of clonal reproduction), known to generate highly non-standard *P*-value distributions are undertaken and finally real population genetics data are analysed. In these cases, all procedures appear, more or less equally, very conservative, though SGM seems slightly more conservative.

**Conclusion:**

Based on our results and those discussed in the literature we conclude that the generalised binomial and Stouffer's *Z *procedures should be preferred and *Z *when the number of tests is very small. The more conservative SGM might still be appropriate for meta-analyses when a strong publication bias in favour of significant results is expected to inflate type 2 error.

## Background

It may happen that researchers have to take into account the results obtained from different independently handled statistical tests of the same null hypothesis. It is then desirable to combine all tests into a single one in order to make the most accurate decision. This is typically the case when one wants to combine the results from different published articles and obtain a global *P*-value over all the tests for global decision making or, in population genetics studies, when the statistical results from different loci or from different kinds of samples must be combined. For instance, it may be desirable to test for genetic differentiation between males and females, between infected and non-infected hosts from different populations or between parasites collected from different host species sampled in sympatry in different locations. Let *p*_1_, *p*_2_,... *p*_*k *_be the *k P*-values obtained. The question asked becomes: "is the *k *tests series significant as a whole?". Beside the Bonferroni procedure and its sequential derivatives [1-3] that are not appropriate in that matter (but see [4-8]), one procedure, the Fisher's method [9,10], is classically used in the literature to combine these *k P*-values into a single one. As already discussed [11-14] Bonferroni is very conservative, and is inappropriate if the goal is to obtain a global *P*-value and not to identify which *P*-values are significant, which is really a very different question (family wide significance of individual *P*-values). Fisher's procedure was held responsible for being sensitive to deviations from uniformity of the distribution of the partial *P*-values by Goudet [15] who then proposed a randomization procedure to test for symmetry around 0.5 using the geometric mean of *P*-values as a statistic (SGM procedure). Fisher's method was also blamed to suffer from asymmetry by Whitlock who proposed Stouffer's *Z*-transformed test [16]. To quote Rice [17], "while useful in many applications", Fisher's test is "inappropriate when asking whether a set of tests, on balance, supports or refutes a common null hypothesis" as it is the case explored in the present paper. An alternative exists that was first introduced by Wilkinson [18] and first applied (to our knowledge) to population genetics data by Prugnolle et al. [19]. At a given type I error rate *α *of say 0.05, if *k *tests are undertaken under the null hypothesis, it is expected that there are about 5% of *P*-values that should be equal or inferior to 0.05 (by definition). Then an exact binomial test with 0.05 expectation, *k*_0.05_, the number of observed *P*-values not greater than 0.05 in *k *trials, should provide the exact probability that a number as great or greater of significant *P*-values can be observed under the null hypothesis. A generalisation of this simple principle was proposed by Teriokhin et al. [13].

In the present note we describe "Multitest V1.2" that implements this generalized binomial procedure. We propose a performance comparison analysis between Fisher, generalised binomial, SGM and *Z*-transformed procedures on simulated population genetics data with randomisation tests where all tests address the same null hypothesis and are all looking at deviations in the same direction. Finally, the comparison is also undertaken on several real data sets. These procedures were never compared before, especially so with randomisation tests on frequency (proportional) data for which minimum *P*-values are bounded by sample size, genetic diversity and randomisation number.

## Implementation

### Parameters used for the Generalised Binomial Procedure

The different parameters we will use here are the following:

*S*: a series of independent tests;

*k*: the number of tests in *S*;

*α*: the chosen level of significance over all the *k *tests;

*S*_*sorted*_: the *k *tests from *S *sorted in increasing order, *P*_1 _the lowest and *P*_*k *_the highest;

*k*': The number of tests in *S*_*sorted *_that need to be equal or under a given level so that *H*_0 _is rejected at level *α *for *S*;

*α*': the level to which all *P*-values from the first to the *k*^'th ^in *S*_*sorted *_must stay equal or inferior (*P*_*k*' _≤ *α*'), so that *H*_0 _can be rejected at level *α*;

*k*_*α*'_: the number of tests that are significant at level *α*';

: the minimum value required for *α *that leads to reject *H*_0_, for a given *k*' or *α*'.

### The Software

Multitest V1.2 is a Windows application developed with Delphi 5 (1999, Inprise Corp). The algorithm, detailed procedure and the Quick-Basic source can be consulted in [13]. The program (MultitestV1-2.exe), the code (MultiTestListing.txt) and help file (NoticeMultiTestV1-2.pdf) are provided as additional files 1, 2 and 3 respectively (see section Additional files). The philosophy behind the test is that the *k *independent *P*-values of the same null hypothesis *H*_0 _should be distributed according to a uniform distribution with mean 0.5 and limits [0,1]. The software was designed to deal with two distinct situations. In the first situation one chooses *k*', the number of partial significant tests that will define, for a given *α*, the level *α*' at which the *k*' tests need to be significant (i.e. ≤ *α*'), so that *S *is significant at level *α*. For this situation we recommend to always use *k*' = *k*/2 or in any case to define *k*' before anything else is undertaken (*k*' should never be chosen *a posteriori*). In the second situation one chooses *α*' that will determine the required number of tests *k*' that need to be significant (i.e. ≤ *α*'), so that *S *is significant at level *α*. This second situation is particularly useful when the exact *P*-values are unknown and levels of significance are indicated by symbols such as "ns" (not significant), "*" (significant at *α *= 0.05), "**" (*α *= 0.01) and "***" (*α *= 0.001).

While running Multitest you are asked to provide several quantities. The first quantity is the desired level of significance. Classically 0.05 is chosen, but you might be more or less severe, particularly if you are looking for , the "exact" threshold *P*-value for the *k *tests series. The second quantity corresponds to the total number of tests you want to combine (*k*). Then you are asked to choose either to fix *k*', and search for *α*', or to fix the value of *α*', and search for *k*', under the chosen overall significance level *α*. If you choose to fix *k*' then the software will outputs *α*' that should be not greater than *P*_*k*' _(*P*_*k*' _corresponds to the *k*^'th ^of your *k P*-values ranked in increasing order). If *α*' <*P*_*k*' _then *S *is not significant at level *α*. If you choose to fix *α*', the software outputs *k*', the number of tests that must display a *P*-value not greater than *α*'. If *k*' > *k*_*α*' _*S *is not significant at level *α*. The precision can also be chosen (default = 10^-4^). Finally, you are asked to choose an output file where all the results are stored in a text file presented as a table sheet. We advise using the .mul extension but this is left to the user's preference.

Let us see one example as illustration. Let us assume that we obtained the following *P*-values after testing for genetic differentiation between males and females of a given imaginary species from ten different localities (*k *= 10): 0.06, 0.07, 0.08, 0.09, 0.1, 0.2, 0.3, 0.5, 0.5 and 0.6 (please note that none of the tests is significant at *α *= 0.05). We want to obtain the *P*-value =  corresponding to *H*_0 _that there is no differentiation between males and females across the *k*-tests series. We set *α *= 0.05, *k *= 10 and choose to test for *k*' = *k*/2 = 5. From there the result is *α*' = 0.22, meaning the series is significant at *α *= 0.05 if it contains at least five tests with *P*-value not greater than 0.22, which is indeed the case as our fifth smallest *P*-value, *P*_5 _= 0.1. A much lower level of significance *α *can be chosen for the series. Here, the minimal level of significance is in fact ≈0.0017, which outputs *α*' = 0.1008 ≥ *P*_5 _= 0.1. Consequently,  represents the *P*-value (highly significant) over all the *k *tests.

### Evaluating Performances of Combining Procedures with Simulations

All simulations were made under Easypop V 2.01 (Balloux 2006, updated from [20])

#### Simulations of Controlled Null Hypotheses

We simulated 1000 Island models (1000 replicates) with free migration (*m *= 1) of 100 randomly mating populations of 100 monoecious individuals each, 10^-5 ^mutation rate, 20 independent loci with *u *= 10^-5 ^mutation rate into 99 possible allelic states, starting with maximum diversity and for 1000 non-overlapping generations. We then tested for genetic differentiation across populations using a random sample of 20 populations of 50 individuals each. The test used was the *G*-based (log-likelihood ratio) randomisation test [21]. The statistic *G *is computed on contingency table of allelic frequencies from the different subsamples and randomisation based on multilocus genotypes (individuals are permuted across subsamples). For each individual test (each locus) *H*_0 _was "there is no differentiation between populations" or, more specifically, "observed *G*, computed on contingency table of allelic frequencies, is not above 95% of *G*'s generated while randomizing individuals across subpopulations". This test was implemented with Fstat 2.9.3 (Goudet 2002, updated from [22]) that also executes a global test across the 20 loci using the additive property of *G *(e.g. [23]). It thus provides a "true" *P*-value that takes into account the information from all loci, weighted with sample sizes and allelic frequencies. For each replicate (1000 simulations) we combined the 20 tests across the 20 loci with the different methods. Note that in Genepop [24,25], Fisher's method is used to combine *P*-values across loci. Please also note that the tests are not *G*-tests but randomisation tests using *G *as a statistic. The *P*-values obtained are thus unbiased estimate of exact *P*-values [26]. This test was deeply investigated [21] and is expected to generate "standard" *P*-value distributions: uniform under *H*_0 _and progressively skewed to lower *P*-values under increasing deviation from *H*_0_. It was undertaken to test and compare the correct behaviour of the different procedures under a realized null hypothesis.

#### Simulations with Controlled Alternative Hypothesis

We chose the randomisation test of linkage disequilibrium (LD) between paired loci of Fstat 2.9.3. Citing Fstat 2.6.3 help file, this option allows testing the significance of association between genotypes at pairs of loci in each sample. The statistic used to test the tables is the log-likelihood ratio *G*-statistic or, more accurately, the only part of this statistic that changes when randomising tables:

where *x*_*ijkl *_represents the number of individuals in the sample with genotype *ij *at the first locus and genotype *kl *at the second locus and where *n *and *m *are the number of alleles at the first and second loci respectively. The *P*-value of the test is obtained as follows. Genotypes at the two loci are associated at random a number of times and the statistic is recalculated on the randomised data set. The *P*-value is estimated as the proportion of statistics from randomised data sets that are larger or equal to the observed. An overall sample statistic is obtained by summing the *G*-statistics overall samples. The overall test is obtained by comparing this overall statistic with that obtained from randomised tables (randomisation occurring of course only within samples). The advantage of this test is that each sample is weighted by its "information" content. The *P*-value in a sample where the two loci are nearly monomorphic (probably very close to 1) should not be given the same weight as a *P*-value from a sample where the two loci are very polymorphic and hence the significance of genotypic association can be thoroughly tested. It thus provides a "true" *P*-value that takes into account the information from all subsamples, weighted with sample sizes and allelic frequencies. LD was chosen because it is probably the population genetics test that generates the most non-standard *P*-value distributions (e.g. U shaped) (as suggested from [27] and confirmed in the present study), thus the closest to natural imperfect data. For all simulations, parameters were 10,000 non-overlapping generations, in an Island model with *n *= 50 subpopulations, *N *= 500 individuals per subpopulation, *m *= 0.001 migration rate, two loci with *u *= 0.00001 mutation rate with 99 possible allelic states. All simulations were replicated 30 times. Alternative hypotheses of increasing strength were obtained by increasing the clonal rate *c *= (0, 0.1, 0.2, 0.3, 0.5, 0.6, 0.7, 0.8, 0.9, 0.95) that generates a corresponding increase in LD between loci [27]. For all simulations 20, 10 or 5 subpopulations of 20 individuals each were sampled, in order to get different values for *k*. Some simulations ended with a few less than *k P*-values because some tests were not feasible in some subpopulations (no polymorphism at one locus). Please note that though the strength of deviation from *H*_0 _is controlled for, *H*_0 _can itself never be simulated. A full independence between loci would require an infinite population size with free recombination for an infinite number of generations. Thus, a signal (even very weak) is expected even with random union of gametes (*c *= 0).

#### Procedures to Combine the *k P*-Values

The binomial probability  was looked after with Multitest V1.2. Note that *α*' is bound to 0.5. When *P*_*k*' _> 0.5, increasing *α *(to get an "exact" *P*-value) invariably outputs *α*' = 0.5. In such cases we simply used the actual value *P*_*k*' _as the global *P*-value. This has no incidence on the results presented in the present paper as we only were interested in  ≤ 0.05 *P*-values.

Fisher's procedure is simply obtained by a Chi-square test with 2×*k *degrees of freedom on the quantity:(1)

The SGM procedure was implemented by the eponym computer program kindly provided by J. Goudet. It uses a randomisation procedure to test the symmetry around 0.5 of the geometric mean of the *k P*-values.

For Stouffer's *Z *transform test, each *P*-value *p*_*i *_is transformed into its standard normal deviate *Z*_*i*_, which, for instance, can be obtained by the normal inverse function of Excel™, with a maximum value of 0.9999 for *p*_*i *_when *p*_*i *_= 1 (i.e. the maximum expected accuracy with 10000 randomisations).

*Z*_*i *_is used for the computation of the statistic *Z*_*s *_[16]:(2)

*Z*_*s *_is then compared to the normal standard distribution (e.g. NORMSDIST(*Z*_*s*_;0;1) in Excel).

A logistic regression exploring the model (*p*_*i *_≤ 0.05) ~*c + k + Method + k*: *Method + Constant *was finally undertaken under S-Plus 2000 Professional release 3 (MathSoft Inc), where *p*_*i *_≤ 0.05 means "significant at the 0.5 level is true", *c *is the clonal rate (with which LD is expected to increase quickly), *k *the number of tests to be combined, *Method *the kind of procedure (Fisher, Binomial, SGM or *Z*) and : stands for "interaction" between parameters. A stepwise procedure was used to select for the best model following the Akaike Information Criterion and remaining parameters tested with a Chi-square.

#### Real Data Sets

Four data sets were used: two data sets on mussel (*Mytilus galloprovincialis*) allozymes from [28] and [29]; one data set on schistosome flukes (*Schistosoma mansoni*) microsatellites [19] and one data set on the opportunistic fungus *Candida albicans *allozymes [30]. We undertook LD tests on these data to compare natural results to our simulations using examples where the exact *G*-based test was significant as a signature for false *H*_0_.

Finally we used some non LD-based real datasets to give examples of application when no global test is available. Two data sets are from [15] (key innovation and rate of speciation in different taxonomic groups) for viviparity in fishes [31] and branch length in angiosperms [32] where contradictions were found between Fisher and SGM procedures and where publication biases may interfere with final results. Two data sets concern examples of combination of non parametric correlation tests: one data set studies the correlation between limpet abundance and cockle shell size on which they settled in New-Zealand shores [33] and one data set examines the correlation between the presence of two pathogenic bacteria in Tunisian cattle individuals [34]. A fifth data set combines test for bottleneck signatures (severe population reduction) on population genetics data in wild rusa deer populations from New-Caledonia [35]. The last data set concerns the results obtained on the relatedness between male and female cattle ticks found as pair on different hosts and different farms in New-Caledonia [36].

## Results and Discussion

### Simulations of Controlled Null Hypotheses

The global *G *outputs 44 significant tests at *α *= 0.05 (out of 1000 replicates), and Fisher, Binomial, SGM and *Z *outputted 51, 48, 42 and 45 significant tests respectively. None of these values significantly deviates from the expected 5% (Exact binomial test, *P*-value > 0.27).

To conclude, all procedures are fine under *H*_0 _and give rather equivalent results.

### Simulations with Controlled Alternative Hypothesis

**Figure 1:**
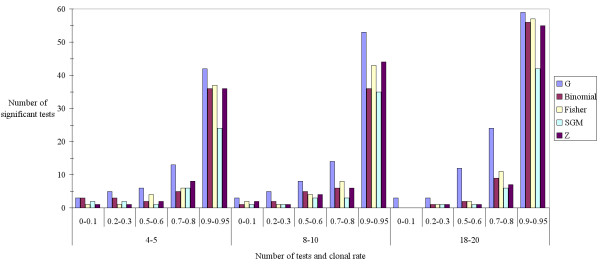
**Number of significant linkage disequilibrium tests (*α *= 0.05) as a function of increasing number of tests combined and increasing global linkage across all loci (clonal rate increase): for the most accurate test (*G*) and different combining procedures (Fisher, Binomial, SGM and *Z *as defined in the text)**. The number of tests was 60 for each bar (30 replicates × 2 modalities for each clonal rate and number of tests) (see text for more details on simulations).

The first important result, though beyond the scope of the present paper, is that the power of LD test is weak as it can be observed from Figure 1. A substantial amount of significant tests only arise for *c *= 0.9 (90% clonal reproduction). The second result is that, in case of non-standard *P*-value distributions, combinatory procedures are very conservative. The third observation resulting from Figure 1 is that all procedures perform more or less equally at least for these tests and simulations. The logistic regression kept *c *with the strongest (and expectedly positive) impact, *Method*, because SGM seemed apparently less powerful than the others and *k *with a positive effect. Globally, *G*, Fisher, Binomial SGM and *Z *respectively displayed 253, 229, 215, 170 and 214 significant tests. The slightly lower power of SGM probably comes from the fact each time a *P*-value is close or equal to unity, it becomes almost impossible for the procedure to output a significant result, even when a substantial proportion of tests in the *S *series are very small. In fact this test is especially conservative in case of U-shaped *P*-value distributions. It was indeed designed for combining published *P*-values on the same null hypothesis, in which case a publication bias is expected and thus for which more weight for non-significant results may be desirable (see also [16]). Because of the nature of LD tests, when *H*_0 _is far from true, U-shaped distributions are likely to occur because in some populations polymorphism will be insufficient at one locus, leading to very high *P*-values and to very small *P*-values in subpopulations where polymorphism is high enough. This is also likely to occur often in many population genetics data sets where the power of the different tests in a series will rarely be identical and most of the case highly variable because of uneven sample sizes (not explored here) and variable genetic diversity across sub-samples.

### Real Data Sets

For LD tests, only independent series (no locus repeated) for which the global *G*-based test provided a significant *P*-value are presented. A glance at Table 1 confirms the lack of power of combining procedures and that the different procedures do not necessarily lead to the same decision, hence the choice is far from neutral. This general tendency is confirmed with the non LD-based data sets (Table 2). For literature based data, SGM interestingly outputs non-significant results in opposition to other procedures. Here, publication bias might be interfering and the most conservative SGM may be more appropriate, providing the several *P*-values close to unity are not due to low power tests. It may happen that some tests were made in samples verifying *H*_1 _and others *H*_0_. Mixing 10 *P*-values from our simulated *H*_0 _with 10 *P*-values from LD tests on our simulations with the maximum expected signal (*c *= 0.95) did not spectacularly dropped the proportion of significant global tests but for the binomial (100% detection to 50% detection). There is indeed no reason that such situations would generate more *P*-values very close to 1 than expected under full *H*_0 _and such phenomena are not expected to affect SGM much.

**Table 1:** Comparison between different combinatory tests for real data with the exact multisample test *G *for linkage disequilibrium (More details can be found in the text).

Organism	Locus pair	*k*	*G*	Fisher	Binomial	SGM	*Z*	References
Mussel	MPI vs ESTD	12	0.0079	0.0915	**0.0180**	0.9330	0.8620	[28]
Mussel	PEPA vs PEPD	5	0.0110	**0.0357**	0.0904	**0.0330**	**0.0154**	[29]
Schistosome	F vs L28	27	0.0039	0.2841	0.3105	0.9865	0.9542	[19]
Fungus	HK2 vs FK	5	0.0001	0.0539	0.0821	**0.0355**	**0.0164**	[30]
Fungus	G6PD vs MPI	4	0.0008	0.0765	0.2630	0.0570	**0.0291**	[30]
Fungus	HK1 vs GPI	2	0.0194	0.0991	0.1908	0.2479	0.0673	[30]

*k*: number of tests combined

Significant combined *P*-values are in bold

**Table 2:** Non LD-based real data sets presenting different cases where combining probabilities methods can be applied.

*H*_0_	*k*	Fisher	Binomial	SGM	*Z*	References
No association between viviparity and number of species in fishes	10	**0.0446**	**0.0081**	0.1804	0.1070	[31]
No association between branch length and number of species in angiosperms	39	**0.0065**	**0.0216**	0.3073	0.1311	[32]
No association between shell size and limpets abundance on cockle	3	**0.0001**	0.0589	**0.0005**	**0.0001**	[33]
Random co-occurrence of *Theileria annulata *and *Anaplasma marginale *in cattle	2	**0.0039**	0.1240	**0.0050**	**0.0024**	[34]
No bottleneck in rusa deer wild populations with the SMM model of mutation.	8	**0.0298**	**0.0488**	**0.0155**	**0.0170**	[35]
Assortative pairing of female and male cattle ticks on their host	20	0.3417	0.1424	0.1644	0.1928	[36]

SMM: Stepwise Mutation Model (applies to microsatellite loci)

Significant combined *P*-values are in bold

## Conclusion

"Fisher's testing procedure represents a test against broad alternatives. It specifically tests whether *at least one *component test is significant, and can yield a significant combined test statistic when the component tests, on balance, strongly support *H*_0_. This is an undesirable characteristic when asking whether a group of tests collectively supports the same *H*_0_" [17]. Bonferroni (and its sequential derivatives) is specifically designed for identifying which tests are significant in a series or, to phrase it in a more statistical way, it is designed to test family wide significance of individual *P*-values [17]. To illustrate this, a 100 tests series with a single *P*-value = 10^-9 ^and where the remaining 99 tests follow a uniform distribution with mean 0.5 will output 0.045 with Fisher, 10^-7 ^with Bonferroni, 0.38 with the generalised binomial, 0.27 with Stouffer's *Z *and 0.26 with SGM. Here, if the alternative hypothesis is that a signal exists across *all *tests, generalised binomial, Stouffer's *Z *or SGM are more appropriate, knowing that a strong lack of power will be met each time the *S *series will deviate from uniformity (e.g. U-shaped). If *H*_1 _is "there is at least one significant test" then Fisher and even Bonferroni are more appropriate and will provide a very different result (hence the importance of a priori defining *H*_1_). It is noteworthy signalling that a weighted version of *Z*, more powerful, was also proposed [16]. For population genetics data, weighting is a complex interaction between sample sizes and allelic frequencies, but an interesting trail to follow may come from there. Note that we did not study the effect of uneven sampling sizes that might also change some conclusions. For published *P*-values combination, the conservative SGM procedure might be preferred when a publication bias is suspected, but users should be aware that this test will always be very conservative when one or few tests are close to unity. Choosing which procedure should be preferred will require further more sophisticated approaches and thus stays a matter of personal convenience. Nevertheless, one advantage of the binomial approach is that it can work even when the exact values of probabilities are unknown but only their significance at a given level, a property not shared by any of the other procedures that all require numerical inputs. One disadvantage of the generalised binomial is its lack of symmetry, especially so when the number of tests is small (or very small). For instance, when *k *= 2 with *P*_1 _= 0.02 and *P*_2 _= 0.98, the generalised binomial will output *P*-value = 0.0397 instead of 0.5 (as obtained with Stouffer's *Z*). In such very particular cases (very small number of tests), it will probably be wiser using Stouffer's *Z*.

## Availability and Requirements

**Project name**: MultiTest

**Project home page**: http://gemi.mpl.ird.fr/SiteSGASS/SiteTDM/Programs

**Operating systems**: Windows (XP, Vista)

**Programming language**: Delphi 5.

## Abbreviations

*H*_0_: Null hypothesis; *H*_1_: Alternative hypothesis; LD: Linkage disequilibrium between loci; SMM: Stepwise Mutation Model (applies to microsatellite loci).

## Authors' contributions

TDM wrote the program, undertook and analysed the simulations, re-analysed real data sets and wrote the paper. JFG contributed in the guidance of the project and corrected the manuscript. ATT wrote the algorithm, contributed in the guidance of the project and corrected the manuscript. All authors read and approved the final manuscript.

## Supplementary Material

Additional file 1 The executable program file, "MultitestV1-2.exe". Click here for file

Additional file 2 The source code in text format, "MultiTestListing.txt". Click here for file

Additional file 3 The help file, in Adobe Acrobat format, giving all the instructions needed to use the program, "NoticeMultiTestV1-2.pdf". Click here for file
