# Supplementary material for: MultiTest V.1.2, a program to binomially combine independent tests and performance comparison with other related methods on proportional data
Source: BMC Bioinformatics. 2009 Dec 23;10:443. doi: 10.1186/1471-2105-10-443 (PMC2811122; doi:10.1186/1471-2105-10-443)
Supplement: Additional file 3 — The help file, in Adobe Acrobat format, giving all the instructions needed to use the program, "NoticeMultiTestV1-2.pdf". [file 1471-2105-10-443-S3.PDF]

Multitest V1.2 Notice

May 2009

Contents

The problem

You choose to fix *k*'

You choose to fix alpha'

Some hints

How to cite Multitest

References

The problem

Say you want to combine the series *S*=0.45, 0.1, 0.15, 0.2, 0.3, 0.35, 0.2, 0.4, 0.5, 0.1 of *k*=10 independent *P*-values corresponding to the same null hypothesis H_0. You want to know if it is possible to obtain such a series if H_0 is true and use a procedure different from Fisher's (Fisher, 1970), more concervative SGM (Goudet, 1999) or Stouffer's *Z* transform test (Whitlock, 2005). The binomial approach was first introduced by Wilkinson (Wilkinson, 1951) and the generalised version by Teriokhin et al. (Teriokhin *et al.*, 2007). To compute the probability of occurrence of *S* under H_0 (a global *P*-value) with the generalised binomial procedure implemented in MultiTest you first need to sort *S* into *S'*=0.1, 0.1, 0.15, 0.2, 0.2, 0.3, 0.35, 0.4, 0.45, 0.5 and then launch Multitest. You obtain the following frame:

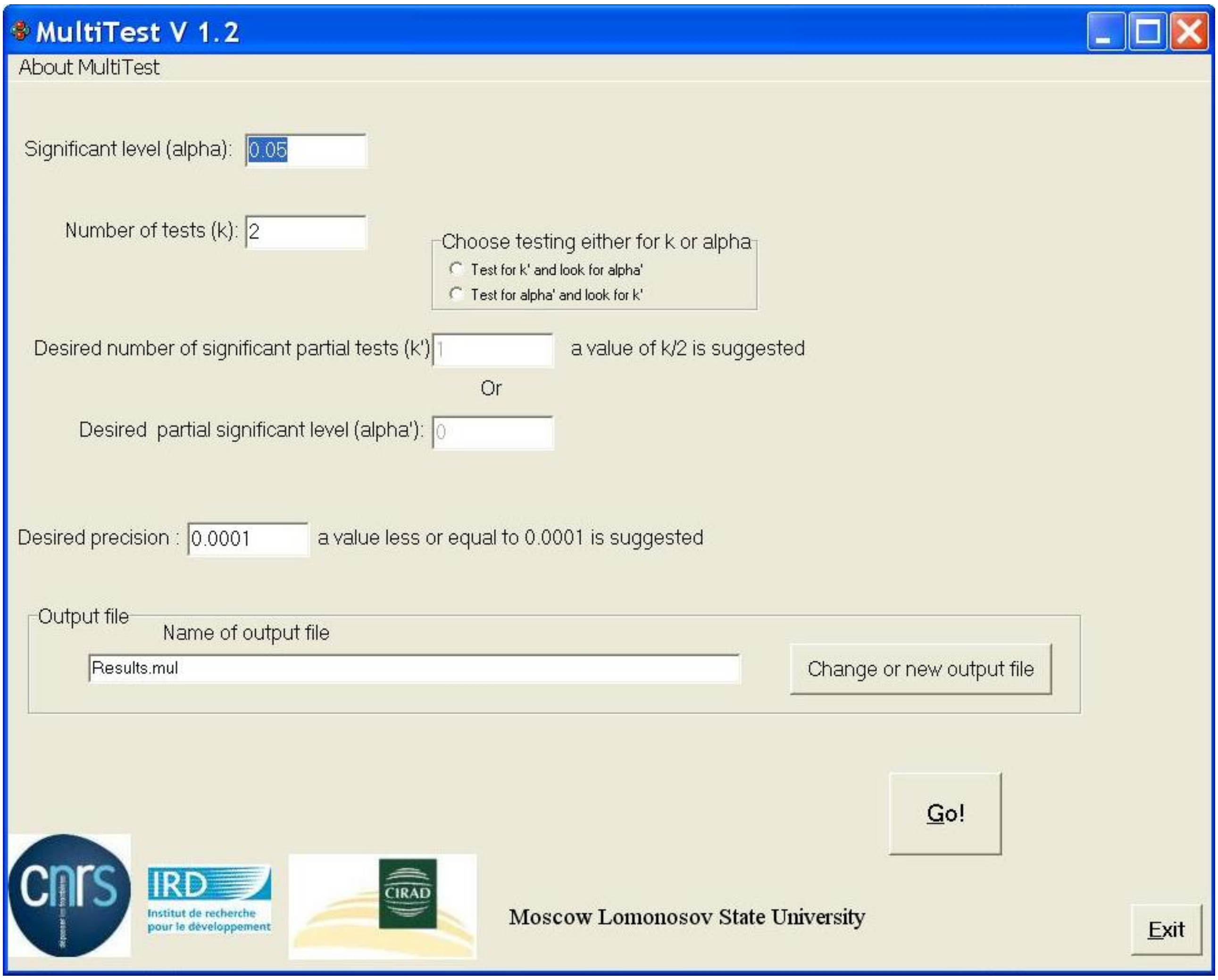

You do not need to change the significance level (0.05) now. Give directly the number of tests you want to combine (*k*), which is here 10. Then you choose either (i) to fix *k*', the number of tests that need to stay below a level that must be found (alpha'), or (ii) to fix alpha' the minimum level of partial significance and look for *k*' the number of tests in *S* that must be equal or below alpha'.

You choose to fix *k*'

The program proposes you *k*/2, which we suggest to always use, unless you have a good **a priori** reason to choose another value. Here, *k*/2=5. The desired precision can be let as it is as a 10^{-4} precision is far enough for most kind of naturally obtained *P*-values. You can choose the name and location of the file where the results will be stored by clicking the button "Change or new output file".

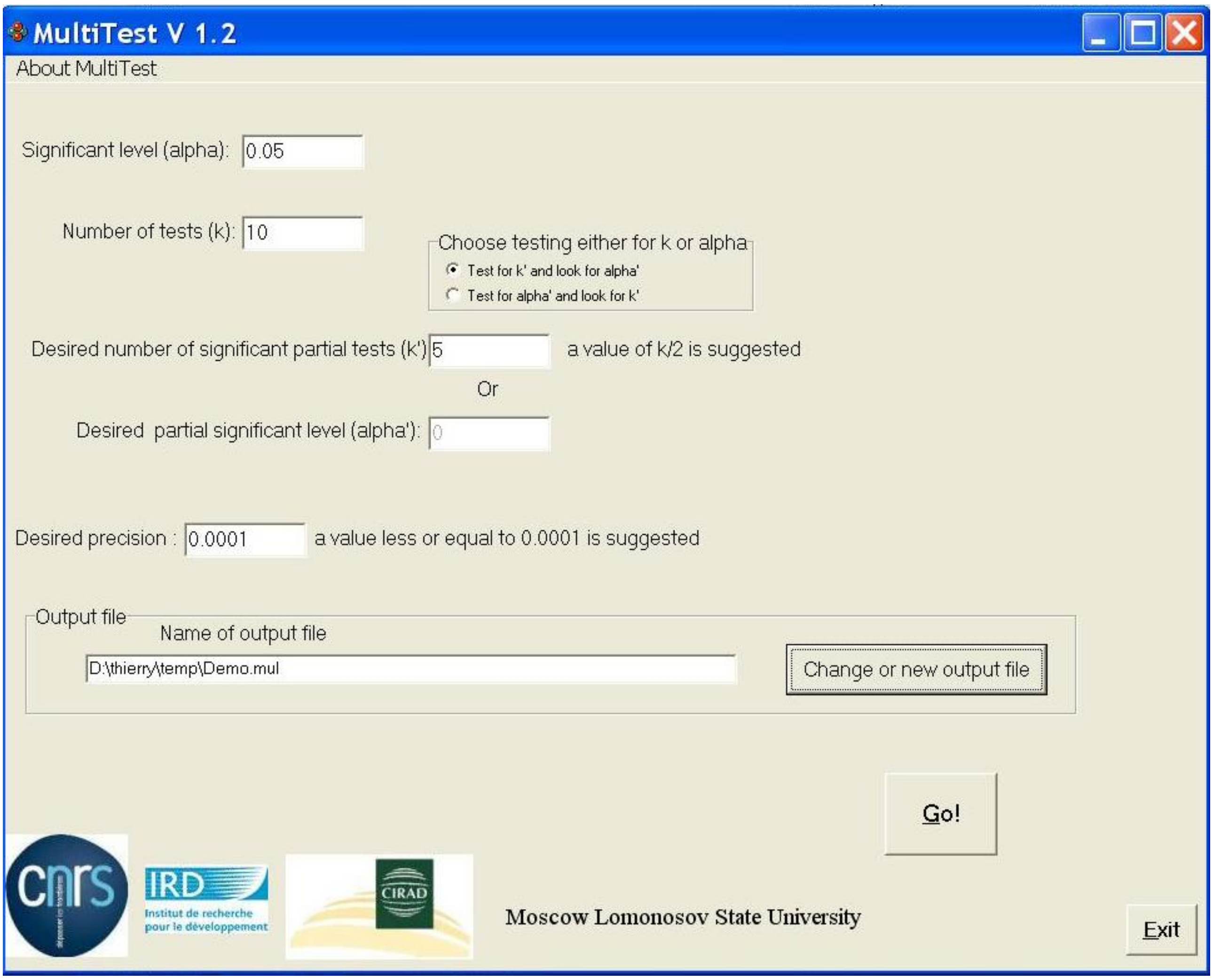

Note that the extension .mul is just here as a suggestion but you can choose any extension you want. Please note that you need to type an extension if you want one.

Then press the Go! button or "G" or "g" on your keyboard.

A small message frame appears.

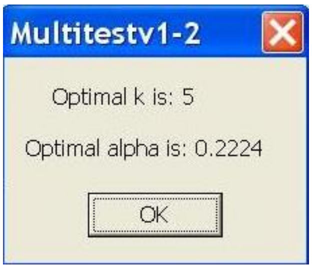

It recalls the *k*' you chose and the resulting alpha', which is here 0.2224. After reading this message you can close this little window by clicking on the button (OK). This message means that if five *P*-values of *S* are equal or below 0.2224 then the series is significant at level 0.05. This is indeed the case as the fifth lowest *P*-value of the series (easy to see in *S*') is $P_{k'}=P_5=0.2$. We can stop there or decide to find the threshold alpha for which alpha' is indeed equal to $P_{k'}$ (here 0.2).

For this, you only need to change the value of alpha (0.05) into a lower value (say 0.045). This will output an alpha' of 0.2165. A lower value for alpha, 0.04, gives 0.2101. Alpha=0.35, 0.2032 etc… until the lowest alpha is found, which is 0.0328 with an alpha'=0.2. From there you can say that *S* is significant with *P*-value=0.0328.

You choose to fix alpha'

The frame looks like this:

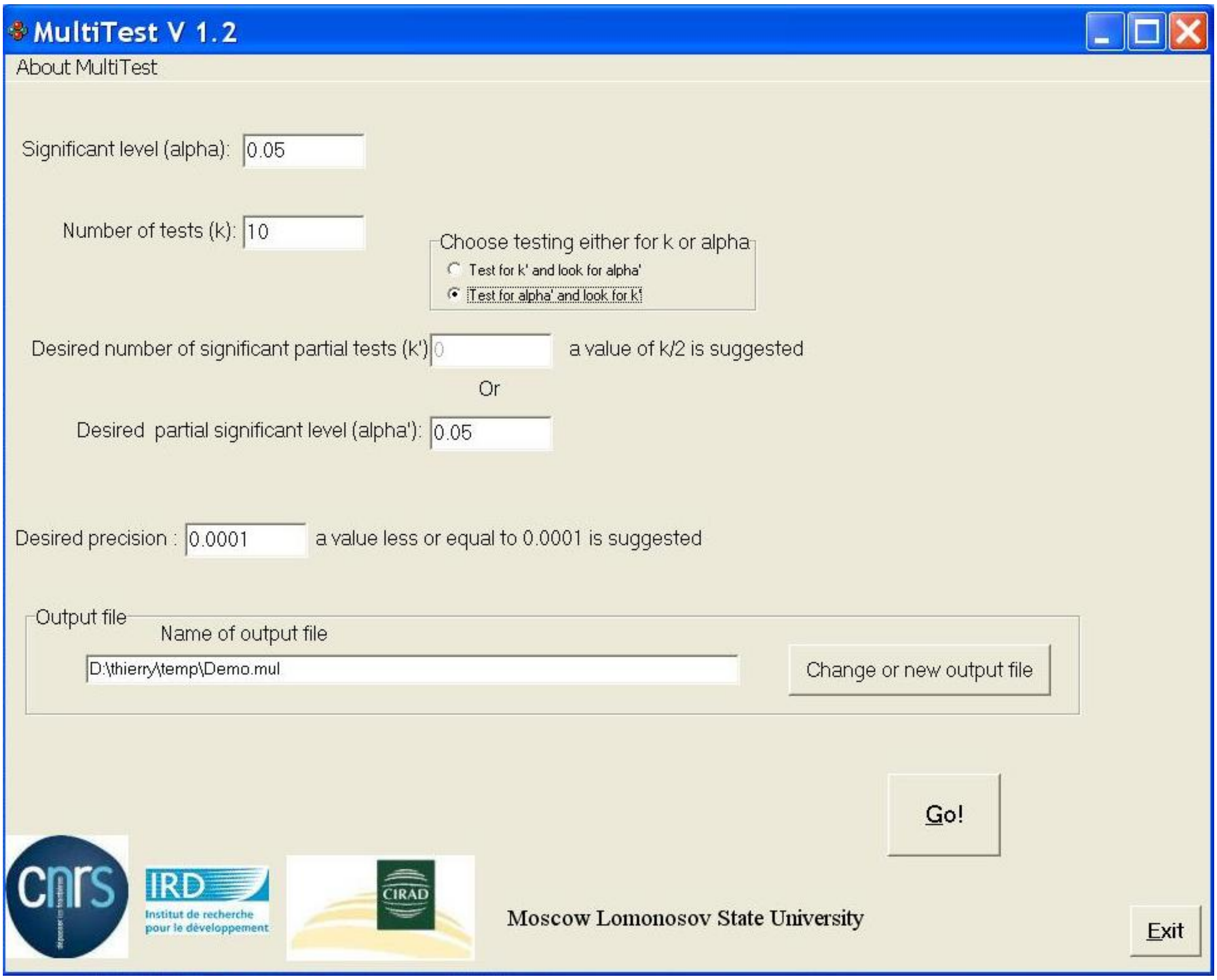

You can let alpha' at 0.05 and click "Go!". The message will be:

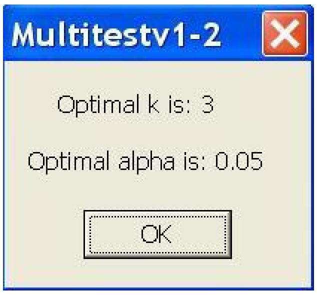

It means that three tests with a P-value≤0.05 are needed for the series to be significant at level alpha=0.05. Here, none of the P-value is below this threshold. If we stay there we must accept H_0. We could however choose a higher alpha'. We know that with alpha'=0.2 the series will indeed be significant. Nevertheless, this is only because we have undertaken the first process that we know

that, and alpha' should not be chosen a posteriori. This is why we advise to always use the first method with $k'=k/2$ when the P-values are known with enough precision and to use the second method when the individual P-values are not known with precision as it is the case when combining the results from different publications where the significance of the tests are often of the form ns, *, ** and ***.

The final results of all processes are stored in the file Demo.mul in the following format:

Alpha	k	k"	alpha"	Precision	alphaOpt	kOpt
0.050000	10	5	0.000000	0.000100	0.222400	5
0.045000	10	5	0.000000	0.000100	0.216500	5
0.040000	10	5	0.000000	0.000100	0.210100	5
0.040000	10	5	0.000000	0.000100	0.210100	5
0.035000	10	5	0.000000	0.000100	0.203200	5
0.030000	10	5	0.000000	0.000100	0.195600	5
0.033000	10	5	0.000000	0.000100	0.200300	5
0.032300	10	5	0.000000	0.000100	0.199200	5
0.032800	10	5	0.000000	0.000100	0.200000	5
0.050000	10	0	0.050000	0.000100	0.050000	3

The first five columns correspond to what you entered and the two last ones to the results printed in the little message window. The program will always write at the end of this table if the output file name and location are note changed.

Some hints

In the case of fixed k', the output alpha' is bounded to 0.5. For instance, the series (0.2, 0.3, 0.4, 0.5, 0.6, 0.7, 0.8, 0.9, 0.9, 1) of 10 tests, with $P_{k/2}$=0.6, will output alpha'=0.4999 for all alpha≥ 0.6229. This means that you cannot get an "exact" P-value in such case. Though it is not a real problem since it only occurs in the non-significant zone, you can just output that P-value≥0.6229 (the limit for k'=5).

When the number of tests to combine is very small (say 2), it is recommended using Stouffer's Z (Whitlock, 2005) instead of MultiTest. This is because the asymmetric nature of the generalised binomial procedure represents a problem when the number of tests is very small hence

the representativeness of *k*/2 is weak. For instance, with P_1=0.02 and P_2=0.98, the generalised binomial will output *P*-value=0.0397 instead of 0.5 (as obtained with Stouffer's *Z*).

How to cite Multitest

In the text:

MultiTest V.1.2 (De Meeûs et al., 2009)

In the reference list:

De Meeûs T., Guégan J.F., Teriokhin A. 2009. MultiTest V.1.2, a program to binomially combine independent tests with a comparison to other related methods on proportional data. Submitted.

References

Fisher RA (1970) *Statistical Methods for Research Workers, 14th Edit* Oliver and Boyd, Edinburgh.

Goudet J (1999) An improved procedure for testing the effects of key innovations on rate of speciation. *Am Nat* **153**, 549-555.

Teriokhin AT, De Meeûs T, Guegan JF (2007) On the power of some binomial modifications of the Bonferroni multiple test. *Zh. Obshch. Biol.* **68**, 332-340.

Whitlock MC (2005) Combining probability from independent tests: the weighted Z-method is superior to Fisher's approach. *J. Evol. Biol.* **18**, 1368-1373.

Wilkinson B (1951) A statistical consideration in psychological research. *Psychol. Bull.* **48**, 156-158.
